# Methylenetetrahydrofolate Reductase Polymorphisms and Risk of Acute Lymphoblastic Leukemia-Evidence from an updated meta-analysis including 35 studies

**DOI:** 10.1186/1471-2350-13-77

**Published:** 2012-09-04

**Authors:** Haigang Wang, Jiali Wang, Lixia Zhao, Xinchun Liu, Wenjie Mi

**Affiliations:** 1 Pharmacy Intravenous Admixture Services, Qilu Hospital, Shandong University, 44 Wenhuaxi Road, Jinan, 250012, China; 2 College of Pharmacy, Shandong University, Jinan, China; 3 Department of Emergency, Qilu Hospital, Shandong University, Jinan, China; 4 Department of Pharmacy, Qilu Hospital, Shandong University, Jinan, China

**Keywords:** Acute lymphoblastic leukemia, Polymorphism, Meta-analysis, 5,10-methylenetetrahydrofolate reductase, Update

## Abstract

**Background:**

5,10-methylenetetrahydrofolate reductase (*MTHFR*) variants, C677T and A1298C, have been reported to be associated with decreased risk of acute lymphoblastic leukemia (ALL). However, results derived from individually underpowered studies are conflicting. We carried out an updated meta-analysis on the association between *MTHFR* polymorphisms and ALL risk.

**Methods:**

Relevant publications were searched through PUBMED and EMBASE databases. The associations between *MTHFR* C677T and A1298C polymorphisms and the risk of ALL were evaluated by odds ratios (ORs). The heterogeneity and publication bias were estimated. Meta-regression analysis was performed to evaluate the potential sources of heterogeneity.

**Results:**

C677T polymorphism was associated with a reduced risk of ALL (allele contrast: OR_RE_ = 0.91, 95% CI: 0.83-0.99). Subgroup analysis showed *MTHFR* C677T variant was associated with decreased susceptibility to ALL in children and Caucasians. Meta-regression showed the logOR for the association between T allele and ALL increased as sex ratio (M/F) in the case group increased (*P* = 0.01). Regarding A1298C polymorphism, no significant association was observed (allele contrast: OR_RE_ = 1.01, 95% CI: 0.91-1.11). There was no publication bias for C677T or A1298C polymorphism.

**Conclusions:**

The present meta-analysis suggests that the C677T polymorphism, not A1298C, in *MTHFR* gene is associated with a decreased risk of ALL, particularly among children and Caucasians subjects. Our findings suggest that the influence of the C677T polymorphism on ALL susceptibility is modified by sex ratio in cases (M/F). Since folate intake may be a possible confounding factor, including this factor in future prospective studies is warranted. Further meta-analysis studies should be at least stratified for folate levels and gender to give more powerful and informative results.

## Background

Acute lymphoblastic leukemia (ALL) is a malignant neoplasm of the lymphocyte precursor cells or lymphoblasts. This hematologic malignancy accounts for 75% of pediatric leukemias and 20% of adult leukemias, with an early peak incidence at 2 to 5 years of age followed by a second peak after age 50 years[1-3]. To date, the cause of ALL remains largely unknown and is likely to involve a complex interaction between genetic susceptibility and environmental exposure [4].

Folate metabolism plays an essential role in both DNA synthesis and cellular methylation reactions (e.g., DNA methylation). The enzyme 5,10-methylenetetrahydrofolate reductase (MTHFR) is a key player in folate metabolism, which irreversibly catalyzes the reduction of 5,10-methylenetetrahydrofolate (5,10-methylene THF) into 5-methyltetrahydrofolate (5-methyl THF), the predominant circulatory form of folate (Additional file 1: Figure S1) [5]. The *MTHFR* gene, containing 11 exons and 10 introns, is located on the short arm of chromosome 1 (1p36.3) [6,7]. Two common polymorphisms in the *MTHFR* gene, C677T (rs1801133) and A1298C (rs1801131) contribute to reduced enzyme activity and disturbance in folate metabolism. Severe enzymatic activity deficiency results in hyperhomocysteinaemia and is linked to increased risk of neural tube defects and vascular diseases[8-11].

*MTHFR* variants have been reported to be associated with reduced risk of ALL. However, results derived from individually underpowered studies are conflicting. Here, we reevaluate the association between *MTHFR* polymorphisms and ALL in a more comprehensive meta-analysis, providing better power to detect small effect size and performing more detailed analysis on the effects of *MTHFR* C677T and A1298C variants on ALL risk.

## Methods

### Selection criteria and identification of studies

We conducted a comprehensive search of PUBMED and EMBASE databases for publications on the association between *MTHFR* C677T and/or A1298C variant(s) and ALL, using the following search terms: methylenetetrahydrofolate reductase or MTHFR; leuk(a)emia, acute lymphocytic or acute lymphoblastic; and gene, polymorphism or genetic variant. The latest searches were undertaken on Oct 3, 2011. All relevant articles identified through the search were scanned on the basis of title and abstract. The articles that clearly did not meet the inclusion criteria were rejected in the initial screening. The appropriateness of remaining articles for inclusion in the meta-analysis was assessed by reading the full text. All references cited in the studies were reviewed to identify additional publications. We also evaluated previous meta-analysis articles [12-17] and manually searched bibliographies to ensure that any relevant but previously omitted articles were included in the present study.

The meta-analysis included case–control, cross-sectional and cohort studies that met all of the inclusion criteria as follows: (i) provided cases of ALL and control subjects without hematologic or other malignancies; (ii) provided relevant data to calculate the odds ratio (OR); (iii) published in English language journals. Case reports, editorials and review articles were excluded. Family-based association studies and genome-wide linkage scans were also excluded for different design considerations. When multiple studies reported on the same population, we used the most recent one only.

### Data collection

For each eligible study, the following data were extracted independently by 2 investigators, using a piloted data extraction form: first author, year of publication, demographics (age, sex, and ethnicity), study design, genotyping method, leukemia characteristics, number of case and control subjects, source of controls and blinding of laboratory workers to participant status. The frequencies of the allele and the genotypic distributions were extracted (if not available, the allele frequencies were calculated from genotypes), for both the cases and the controls. When articles presented data for different ethnic groups, results for the subgroups were considered as separate studies. If raw data could not be extracted for the meta-analysis, we attempted to obtain this information by corresponding with the authors. Discrepancies were resolved by discussion, when necessary, adjudicated by a third reviewer.

### Data synthesis and analysis

The risk of ALL associated with the *MTHFR* C677T and A1298C polymorphisms was evaluated by OR with corresponding 95% confidence intervals (CIs) under allele contrast, dominant model, recessive model and additive model[18]. In addition, subgroup analysis was carried out by ethnicity (Caucasians or East Asians) and study population (adults or children).

The interstudy heterogeneity in terms of degree of association was tested using the Cochran’s *Q*-statistic [19]. If *P <* 0.10, the heterogeneity was considered significant, which was further explored by *I*^2^ statistic. *I*^2^ is expressed as the percentage of between-study variability that is attributable to genuine variation rather than sample error[20]. If there was heterogeneity among studies, we used a random-effect (RE) model to pool the ORs; otherwise, a fixed-effect (FE) model was selected [21].

Given that immunophenotypic subtypes of ALL (B or T-lineage ALL), sex ratio (males vs females, M/F) might modulate the effects of *MTHFR* polymorphisms on ALL risk, and year of publication, journal impact factor (according to the Journal of Citation Report 2010) might lead to publication bias and heterogeneity, we included these factors as covariates in meta-regression. The cumulative and recursive cumulative meta-analysis were performed to demonstrate how evidence concerning the genetic association has evolved over time [18]. The Egger regression test and Begg–Mazumdar test were used to estimate the potential publication bias [22]. Pearson’s χ^2^ test was used to evaluate Hardy-Weinberg equilibrium (HWE) in the control group for all studies. Studies with controls not in HWE or studies not reporting enough information to evaluate HWE were subjected to a sensitivity analysis. Furthermore, we omitted 1 study at a time to assess the stability of results in the sensitivity analysis. Analyses were performed with Stata software (version 10.0; Stata Corporation, College Station, Texas, USA), using two-side *P-*values.

## Results

### Characteristics of the included studies

**Figure 1:**
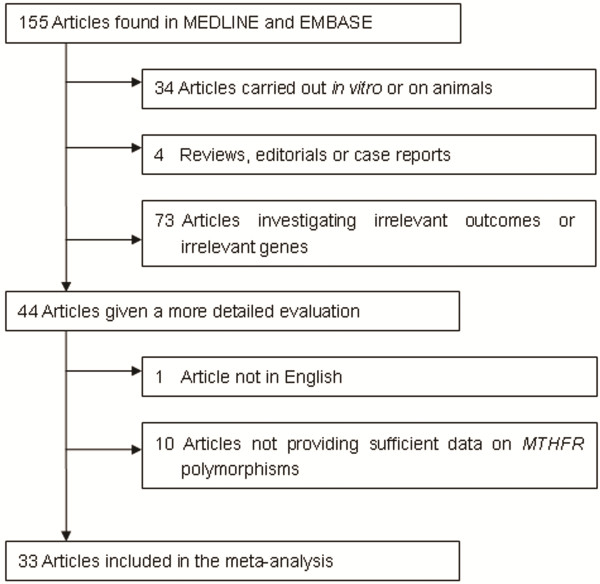
Flowchart describing the process of articles inclusion/exclusion.

The literature search identified 155 articles. After abstract examination, 111 articles were excluded, and 44 articles were retrieved and evaluated against the inclusion criteria. Data from 33 articles[5,23-54] that investigated the association between *MTHFR* polymorphisms and ALL met the inclusion criteria. Figure 1 presents a flowchart for the process of articles inclusion/exclusion, with specification of reasons. All of the articles were in full length except one in letter [32]. Two articles provided separate data for 2 ethnic groups each [33,48]. Thus data were obtained from 35 studies. 

The studies were published from 1999 through 2010 (Additional file 2: Table S1). All the studies were described as case–control in design. Six studies [23,25,28,39,44,46] involved adult ALL patients, 26 [5,24,26,27,30-35,37,38,40,42,43,45,47-54] involved pediatric patients and 3 [29,36,41] involved a mixed population of adult and childhood patients. The identified studies were undertaken in a wide range of ethnicities: 15 [23,27-33,37,38,43,45,47,52,54] providing data on Caucasians, 7 [35,36,39,44,46,48,49] on East Asians (Chinese and Korean), and 13 [5,24-26,33,34,40,42,43,48,50,51,53] on other ethnic origins. Twenty-one studies [5,26,28-31,33,38-41,43-45,49-54] involved general population-based controls and 5 studies [23,27,34,42,45] involved hospital-based controls, 9 studies [24,25,32,35-37,47,48] did not describe the source of their controls. Different genotyping methods were used: polymerase chain reaction (PCR) followed by restriction fragment length polymorphism (RFLP) analysis was used in 26 studies [5,23,24,26,28,29,31,33,34,36-40,44-51,53,54], real-time PCR was used in 8 studies [25,31,32,41-44,52] and allele-specific oligonucleotide hybridization (ASO) was used in 2 studies [27,35]. In most studies, authors reported that the diagnosis of ALL was based on morphologic and immunophenotypic criteria. Eight studies [23,24,28,34,39,47,49,51] stated that the controls were age and gender matched. Only one study [23] mentioned genotyping was performed under blind conditions. Twelve studies provided data for combined genotype distribution of C677T and A1298C variants [23,27,31,32,34,35,38,42,46,50,51] and 4 studies provided analysis of haplotypes for these two variants [39,44,46,53]. In three studies [26,36,39] on C677T and two studies [38,50] on A1298C, the distribution of the genotypes in control group were found to deviate from HWE according to *P* value (*P* < 0.05). Thirty-four studies dealt with C677T, 29 studies dealt with A1298C and 28 studies investigated the two polymorphisms together.

There were 5710 cases and 10798 controls included for the association between C677T polymorphism and the risk of ALL. The frequency (%) of T allele/TT genotype in controls in Caucasians was 35.4/12.9, in East Asians was 40.8/16.0, respectively. The studies provided 5356 cases and 9906 controls for A1298C polymorphism. The frequency (%) of C allele/CC genotype in controls in Caucasians was 31.8/10.1, in East Asians was 18.5/3.3, respectively. Detailed information regarding genotype distribution and allele frequency for cases and controls is available in Additional file 3: Table S2 and Additional file 4: Table S3.

### C677T Polymorphism Associated with A Decreased Risk of ALL

**Figure 2:**
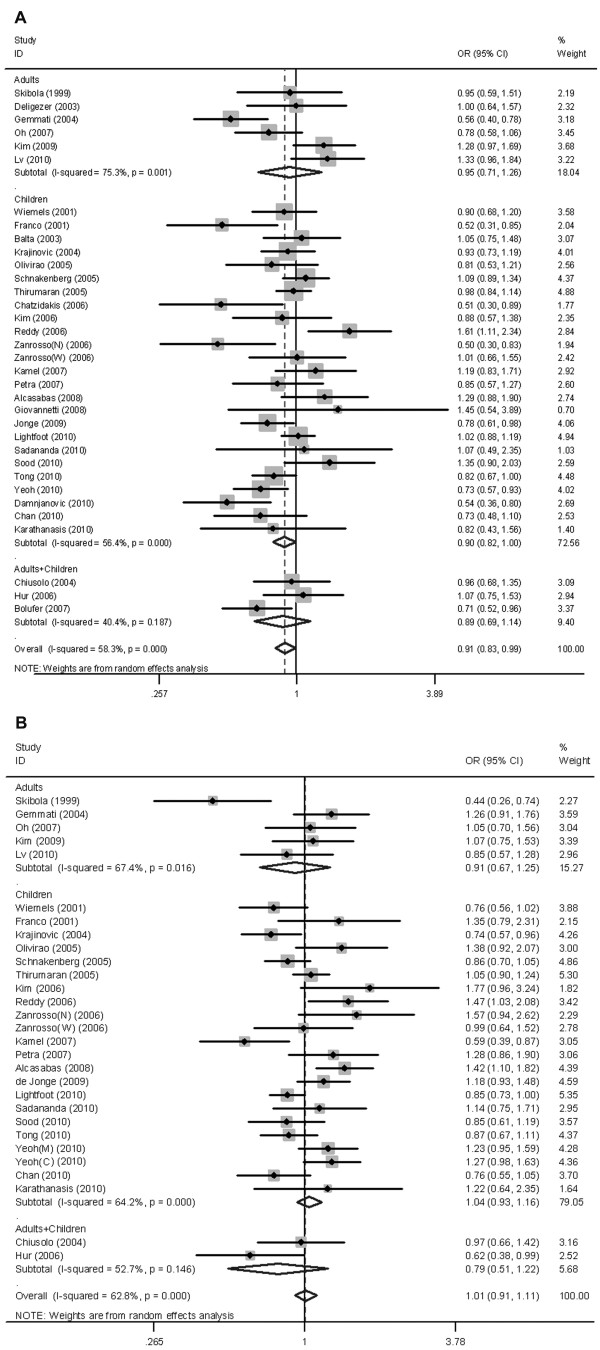
**Random-effect OR estimates with the corresponding 95% CIs for the allele contrast of* MTHFR *C677T and A1298C polymorphisms and the risk of ALL.** (**A**) C677T polymorphism and ALL; (**B**) A1298C polymorphism and ALL. Studies are displayed by ascending order of publication year. The size of the square represents the weight of the corresponding study. C, Chinese; M, Malays; N, non-Caucasians, admixture of Amerindians, Europeans and Africans; W, mainly Brazilians of Caucasian descent.

Table 1 shows the meta-analysis results for C677T polymorphism. Overall, significant heterogeneity between studies was found in all genetic contrasts except recessive model (recessive model: *P*_*Q-Test*_ = 0.13, *I*^2^ = 22%). Marginally significant inverse association was observed in allele contrast, recessive model and additive model (allele contrast: OR_RE_ =0.91, 95% CI: 0.83-0.99; recessive model: OR_FE_ = 0.86, 95% CI: 0.77-0.96; additive model: OR_RE_ =0.80, 95% CI: 0.68-0.95; Figure 2A). After exclusion of the studies lack of agreement of controls with the HWE, there was no significant alteration in the pattern of the results (Table 1). Removal of any one study did not result in movement of the point estimate outside the 95% CIs, suggesting no single study exhibited excessive influence (Additional file 5: Figure S2).

**Table 1:** **ORs and heterogeneity results for the genetic contrasts of* MTHFR *C677T polymorphism for ALL risk**

	**Genetic Model**	**Studies**	***I***^**2**^**, (%)**	***P*, Q Test**^**a**^	**Fixed-effect**	**Random-effect**
					**OR**	**OR**
All	Allele contrast	34	58	0.000	0.92(0.88-0.97)	0.91(0.83-0.99)
	Dominant model	34	58	0.000	0.92(0.86-0.98)	0.90(0.80-1.02)
	Recessive model	33	22	0.129	0.86(0.77-0.96)	0.85(0.74-0.98)
	Additive mode	33	39	0.013	0.82(0.73-0.93)	0.80(0.68-0.95)
All in HWE	Allele contrast	31	61	0.000	0.92(0.87-0.97)	0.90(0.82-0.99)
	Dominant model	31	60	0.000	0.92(0.86-0.99)	0.90(0.80-1.02)
	Recessive model	30	25	0.109	0.85(0.76-0.96)	0.84(0.72-0.97)
	Additive model	30	41	0.011	0.82(0.72-0.92)	0.78(0.65-0.94)
Children	Allele contrast	25	56	0.000	0.92(0.87-0.98)	0.90(0.82-0.997)
	Dominant model	25	60	0.000	0.92(0.85-0.99)	0.90(0.79-1.03)
	Recessive model	24	0	0.479	0.85(0.74-0.96)	0.86(0.75-0.98)
	Additive model	24	23	0.158	0.82(0.71-0.94)	0.80(0.67-0.95)
Adults	Allele contrast	6	75	0.001	0.95(0.83-1.09)	0.95(0.71-1.26)
	Dominant model	6	67	0.010	0.94(0.77-1.14)	0.96(0.68-1.35)
	Recessive model	6	66	0.012	0.93(0.72-1.22)	0.85(0.52-1.40)
	Additive model	6	74	0.002	0.89(0.67-1.19)	0.82(0.44-1.52)
Adults+children	Allele contrast	3	40	0.187	0.88(0.72-1.06)	0.89(0.69-1.14)
	Dominant model	3	52	0.126	0.85(0.64-1.12)	0.86(0.57-1.29)
	Recessive model	3	31	0.236	0.85(0.60-1.20)	0.85(0.55-1.39)
	Additive model	3	30	0.241	0.77(0.52-1.14)	0.79(0.49-1.26)
East Asians	Allele contrast	7	65	0.009	0.92(0.83-1.02)	0.95(0.79-1.15)
	Dominant model	7	60	0.020	0.90(0.78-1.04)	0.95(0.74-1.22)
	Recessive model	7	53	0.047	0.89(0.72-1.09)	0.91(0.66-1.24)
	Additive model	7	60	0.020	0.87(0.69-1.09)	0.90(0.62-1.32)
Caucasians	Allele contrast	15	54	0.006	0.89(0.83-0.96)	0.85(0.76-0.95)
	Dominant model	15	54	0.007	0.88(0.80-0.96)	0.82(0.70-0.95)
	Recessive model	15	27	0.155	0.85(0.73-0.98)	0.82(0.68-0.99)
	Additive model	15	40	0.053	0.80(0.69-0.93)	0.74(0.59-0.92)

All = all of the studies meet the inclusion criteria; All in HWE = all of the studies meet the inclusion criteria except ones with genotype distribution of the controls deviating from HWE; HWE = Hardy-Weinberg Equilibrium.

^a^ Q test is to estimate heterogeneity between studies. *P* < 0.10 indicates significant heterogeneity.

When the analysis was carried out in different age subgroups, the *MTHFR* 677 T variant was associated with a decreased susceptibility to pediatric ALL (n = 25, allele contrast: OR_RE_ = 0.90, 95%CI: 0.82-0.997; recessive model: OR_FE_ = 0.85, 95% CI: 0.74-0.96; additive model: OR_FE_ =0.82, 95% CI: 0.71-0.94; Figure 2A), whereas it did not show reduced risk for ALL in adults (n = 6). Higher degree of heterogeneity was observed in adults in comparison with children subgroup (Table 1). Importantly, recessive model showed the absence of heterogeneity in children (*I*^2^ = 0%). Analysis stratified by ethnicity showed 677 T variant was associated with a significantly decreased risk of ALL in Caucasians under all genetic contrasts (n = 15, allele contrast: OR_RE_ = 0.85, 95% CI: 0.76-0.95). However, no significant association was observed in East Asians (Table 1).

### No association between A1298C polymorphism and risk of ALL observed

Regarding the *MTHFR* A1298C polymorphism, no significant association was observed in any genetic model test when all the 29 studies pooled together (allele contrast: OR_RE_ = 1.01, 95% CI: 0.91-1.11; dominant model: OR_RE_ = 1.02, 95% CI: 0.90-1.16; recessive model: OR_FE_ = 0.99, 95% CI: 0.88-1.12; additive model: OR_RE_ = 1.01, 95% CI: 0.83-1.22; Figure 2B). Heterogeneity between studies was significant in all genetic contrasts except recessive model. Exclusion of studies deviating from HWE did not alter the pattern of results (Table 2). None of the single study exhibited excessive influence on the pooled results (Additional file 6: Figure S3). Subgroup analysis showed evidence for a relationship between A1298C polymorphism and a decreased risk of ALL neither in pediatric (n = 22) nor in adult subjects (n = 5). Stratification taking into account ethnicity also produced no significant results, with a magnitude of effects similar to that found in the main analysis (Table 2).

**Table 2:** **ORs and heterogeneity results for the genetic contrasts of*MTHFR*A1298C polymorphism for ALL risk**

	**Genetic model**	**Studies**	***I***^**2**^**,**	***P*, Q Test**^**a**^	**Fixed-effect**	**Random-effect**
			**(%)**		**OR**	**OR**
All	Allele contrast	29	63	0.000	0.99(0.94-1.05)	1.01(0.91-1.11)
	Dominant model	29	65	0.000	0.99 (0.92-1.06)	1.02(0.90-1.16)
	Recessive model	29	20	0.171	0.99(0.88-1.12)	1.00(0.86-1.17)
	Additive model	29	41	0.012	0.99(0.87-1.13)	1.01(0.83-1.22)
All in HWE	Allele contrast	27	64	0.000	0.99(0.94-1.05)	1.01(0.91-1.11)
	Dominant model	27	66	0.000	0.99(0.92-1.07)	1.02(0.89-1.17)
	Recessive model	27	26	0.113	0.99(0.87-1.22)	1.00(0.84-1.18)
	Additive model	27	44	0.008	0.99(0.87-1.13)	1.01(0.82-1.24)
Children	Allele contrast	22	64	0.000	1.01(0.95-1.07)	1.04(0.93-1.16)
	Dominant model	22	67	0.000	1.01(0.93-1.09)	1.07(0.92-1.24)
	Recessive model	22	26	0.129	1.00(0.88-1.14)	1.00(0.85-1.18)
	Additive model	22	47	0.008	1.00(0.87-1.15)	1.02(0.83-1.25)
Adults	Allele contrast	5	67	0.016	0.96(0.81-1.14)	0.92(0.67-1.25)
	Dominant model	5	68	0.014	0.97(0.79-1.19)	0.92(0.63-1.33)
	Recessive model	5	41	0.150	0.84(0.50-1.42)	0.85(0.37-1.93)
	Additive model	5	51	0.086	0.84(0.49-1.42)	0.80(0.32-2.01)
East Asians	Allele contrast	7	53	0.046	1.01(0.89-1.15)	1.01(0.82-1.23)
	Dominant model	7	63	0.013	1.01(0.87-1.17)	1.01(0.78-1.31)
	Recessive model	7	0	0.730	1.02(0.70-1.50)	1.07(0.72-1.58)
	Additive model	7	0	0.671	1.03(0.70-1.51)	1.07(0.72-1.59)
Caucasians	Allele contrast	12	62	0.003	0.96(0.89-1.04)	0.98(0.86-1.12)
	Dominant model	12	55	0.011	0.95(0.86-1.04)	0.98(0.83-1.15)
	Recessive model	12	42	0.062	0.97(0.82-1.14)	0.97(0.76-1.25)
	Additive model	12	52	0.019	0.95(0.80-1.12)	0.98(0.73-1.30)

All = all of the studies meet the inclusion criteria; All in HWE = all of the studies meet the inclusion criteria except ones with genotype distribution of the controls deviating from HWE; HWE = Hardy-Weinberg Equilibrium.

^a^ Q test is to estimate heterogeneity between studies. *P* < 0.10 indicates significant heterogeneity.

For combined genotypes analysis, we did not observe significant pooled ORs when using 677CC/1298AA combination as a baseline (Additional file 7: Table S4). We did not pool the ORs for haplotypes in the meta-analysis because of the limited data.

### Potential Bias

**Figure 3:**
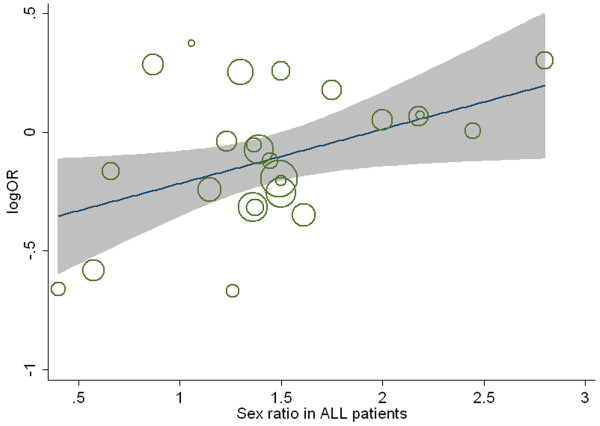
**Meta-regression of sex ratio (M/F) in ALL cases and* MTHFR *C677T genetic effect using allele contrast.** The size of the circles represents the weight of the corresponding study in the meta-regression.

**Figure 4:**
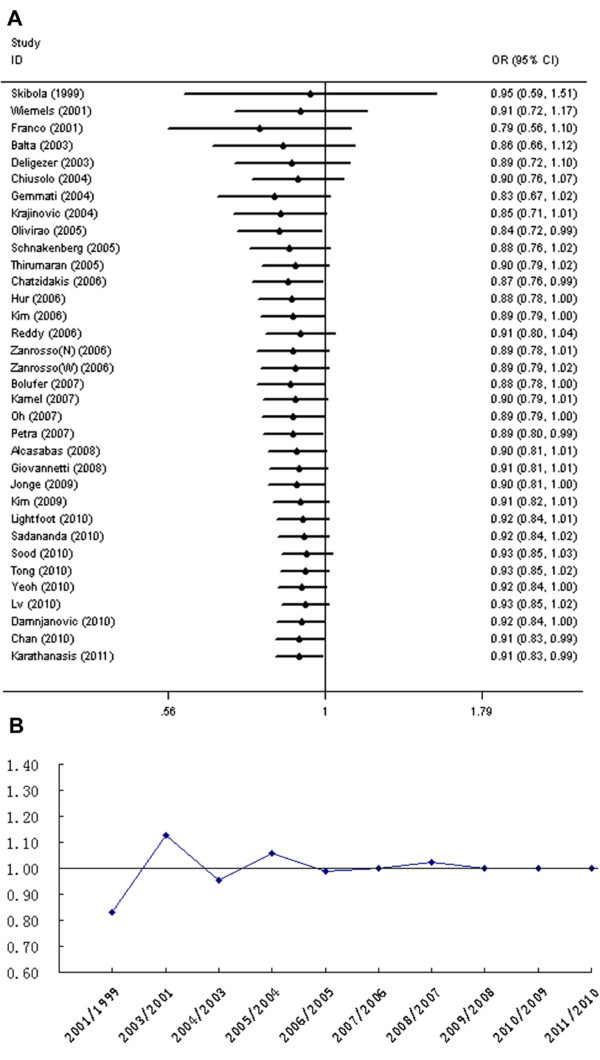
**Cumulative and recursive cumulative meta-analysis for the association between*MTHFR*C677T polymorphism and the risk of ALL.** (**A**) Cumulative meta-analysis; (**B**) Recursive cumulative meta-analysis. Allele contrast was used in cumulative and recursive cumulative meta-analysis. N, non-Caucasians, admixture of Amerindians, Europeans and Africans; W, mainly Brazilians of Caucasian descent.

Meta-regression results indicated a significant correlation between sex ratio (M/F) in ALL cases and genetic effect (n = 25, *P* =0.01), which could explain 28% of the variance (Figure 3), whereas year of publication, journal impact factor, immunophenotypic subtypes and sex ratio in controls did not contribute significantly to between-study heterogeneity (*P* > 0.05). The logOR for the association between T allele and ALL increased as M/F in the case group increased. No covariates modulating the effect of A1298C polymorphism on ALL risk was found (data not shown). Cumulative meta-analysis showed a trend of inverse association between C677T variant and ALL risk as evidence accumulated. Recursive cumulative meta-analysis showed that the relative change in ORs for the C677T polymorphism fluctuated in the beginning years (from 1999 to 2006) and then stabilized at around 1.0 (Figure 4).

No significant publication bias was detected for C677T or A1298C polymorphism by formal statistics (C677T: Egger's test, *P* = 0.42, Begg's test, *P* = 0.51; A1298C: Egger's test, *P* = 0.61, Begg's test, *P* = 0.81; respectively), indicating there is no differential magnitude of effect in large *vs* small studies.

## Discussion

Two meta-analyses published in 2006, focused on the associations between *MTHFR* C677T and A1298C polymorphisms and ALL risk [12,13]. Nevertheless, inconsistent results were obtained between the two studies for C677T and A1298C variants, which might mainly be due to limited number of the included studies and/or different selection criteria[18,55]. In recent two years, another 4 meta-analyses also investigated the associations between *MTHFR* polymorphisms and ALL risk [14-17]. None of the authors carried out analysis in detail which could be done as more studies were available. Additionally, fewer studies were collected in these meta-analyses compared with ours (Additional file 8: Table S5), probably due to insufficient attention in the search strategy or screening process, which might introduce selection bias. Thus, it is not surprising these meta-analyses also failed to give consistent results. Here, the strength of the present analysis is that our study is based on a larger amount of published data and gives comprehensive and intensive information to evaluate the effects of *MTHFR* C677T and A1298C polymorphisms on ALL risk.

It is possible to draw several conclusions from the current data. The results of main analysis support a protective role of the C677T variant in the development of ALL, but not A1298C polymorphism. The stability in sensitive analysis and recursive cumulative meta-analysis indicates that there is sufficient evidence to confirm the beneficial effect of C677T variant in ALL. It has been well documented that *MTHFR* 677 T variant encodes a thermolabile enzyme with reduced catalytic activity and increases plasma homocysteine levels [1,2]. MTHFR enzyme function influences cancer risk in two pathways. Polymorphisms that affect MTHFR enzyme activity decreases the methylation of homocysteine to methionine and in turn the level of S-adenosylmethionine (SAM), resulting in DNA hypomethylation (Additional file 1: Figure S1). This phenomenon can increase the risk of some cancers (e.g. esophageal[56] and gastric cancers[57]). On the other hand, the mechanism proposed to explain the reduced risk of leukemia[23], colorectal carcinoma[58] and other neoplasias is that impaired MTHFR activity, because of polymorphic variation, leads to an accumulation of cytosolic 5,10-methylene THF available for purine and pyrimidine synthesis, thus a lower incorporation of uracil into DNA and a lower cancer risk (Additional file 1: Figure S1). The A1298C polymorphism contributes to less effect on enzyme activity. Decrease in enzyme activity in individuals homozygous for the A1298C polymorphism (30-40% of the wild type) is less than that of C677T homozygotes (60-70% of the wild type). A1298C polymorphism does not seem to be powerful enough to affect plasma homocysteine level, except when accompanied by C677T variant [3,4]. This may partly explain why *MTHFR* C677T variant but not A1298C was found to be protective for ALL.

Our data show that C677T variant plays a protective role in a group of pediatric patients, but has no significantly beneficial effect in adult subjects. There is significant difference between adult and childhood acute leukemia. The most common form of acute leukemia in adults is acute myeloid leukemia (AML), whereas in childhood is ALL[59]. The effect of C677T polymorphism in disease susceptibility may vary depending on folate status. Individuals during the periods of rapid cell division and growth, such as infants and children, have higher folate requirement and are more susceptible to DNA damage as a result of folate insufficiency than adults. In addition, children are not exposed to many environmentally carcinogenic factors, so a diet- and genetic-related etiology of leukaemia is more likely [14].

We observed *MTHFR* C677T polymorphism was associated with a significant reduction of ALL risk in Caucasian subjects, whereas it failed to show any protective effect in East Asians. As the role of the *MTHFR* genotypes in the development of ALL may differ among population origin due to the different dietary customs and racial backgrounds [60], we retrieved the T allele frequency in controls and folate intake in the included studies. There was no obvious difference in the average T allele frequency in controls between Caucasians and East Asians (35.4% and 40.8%, respectively). No study provided data on folate intake. Only one study provided data on the serum folate level in ALL [49]. There may be lower folate status or even folate deficiency in the Asian populations, especially in China where women taking insufficient folate during pregnancy result in a high incidence of infant neural tube defects [61]. Therefore, it is unexpected that the protective effect of C677T polymorphism is not observed in East Asian populations. Importantly, we calculated the pooled results in Caucasian children and East Asian children, separately, and found significant results in both groups (allele contrast: Caucasian children, OR_RE_ = 0.89, 95% CI: 0.79-0.997, n = 11; East Asian children, OR_RE_ = 0.79, 95% CI: 0.69-0.92, n = 3). As we included English studies only in this meta-analysis, the results might be influenced by language bias, especially in non-English ethnicity. We pooled the studies on Chinese children without any restriction on language in another article and found a significant protective effect of *MTHFR* C677T variant on ALL risk (n = 7, data not shown). We cannot exclude the possibility that the difference between Caucasians and East Asians is attributable to chance factors or age composition. Thus, the conclusion derived from such subgroup should be interpreted with caution.

We carried out the meta-regression study to evaluate the potential sources of heterogeneity. For C677T polymorphism, the heterogeneity could be partly attributed to variation in M/F in ALL cases of the original studies. The OR increased as M/F in the case group increased. It was surprising to find OR > 1 in studies with M/F ≥ 2 in case group, indicating the protective effect of T allele disappeared or even turned to the opposite in these studies [25,26,36,50,51]. We limited our analysis to the pediatric patients and observed a similar trend (data not shown). This interesting result motivated us to check three studies providing data on the separate sex group [26,34,46]. Belta et al.[26] and Reddy et al.[34] showed the CT/TT genotypes were more frequent in male than female cases, but Lv et al.[46] did not show any difference on the CT/TT genotypes distribution between male and female cases. The ORs in the male group were also not consistent, with OR < 1 in Reddy’s study and OR > 1 in the Lv’s study. Because of the discrepant results, we could not give the conclusion whether the genetic effects are different or not in separate gender here. It has been reported gender difference exists in ALL susceptibility and ALL is more common in males of all age group, despite the underlying mechanisms for sex difference remain unknown[62]. Our study showed sex ratio in case group modified the C677T variant effects with regard to ALL risk. However, this result is preliminary and deserved further investigation stratified for gender.

Gene-environment interaction between the *MTHFR* genotypes and dietary folate intake has been documented in previous studies concerning colorectal cancer, which might alter the effects of the polymorphic variants [5,6]. With respect to the risk of ALL, epidemiological studies proved a protective effect of maternal folate supplementation during pregnancy against childhood ALL[63,64]. As mentioned above, however, no studies to date described the effect of folate status on the association between *MTHFR* polymorphic variants and ALL susceptibility. Conflicting results among studies may be due to the lack of information on folate status. Other enzymes involved in folate metabolism, including methionine synthase (MS), thymidylate synthase (TS) and serine hydroxymethyltransferase (SHMT), may regulate intracellular folate metabolism. The variant forms of these enzymes may be associated with the risk of ALL and interactions between these candidate genes may exist. Three included studies described the interactions between *MTHFR* polymorphisms and other gene variants. Jonge et al. [45] found the *MTHFR* C677T and *NNMT* C-151 T variants interacted to decrease the risk of pediatric ALL. Petra et al. [38] reported the *MTHFR* C677T, *MS* A2756G and *MTRR* A66G interaction was associated with a reduced risk of pediatric ALL, whereas Gemmati et al. [28] did not detect a significant interaction between *MTHFR* C677T and *MS* A2756G polymorphisms. No further analysis on these interactions in our study was performed due to limited number of reports on each item.

## Conclusion

In conclusion, the present meta-analysis suggests that the C677T polymorphism in *MTHFR* gene is associated with decreased susceptibility to ALL, and indicates a lack of positive relationship between A1298C polymorphism and ALL. The C677T variant plays a protective role in pediatric patients and Caucasian subjects. M/F in cases could modulate the influence of the C677T polymorphism on ALL susceptibility. Although more than 30 genetic association studies are included in this meta-analysis to draw relative safe conclusions, it is also worth mentioning that several interesting but unsolved issues are raised from current meta-analysis and thus further studies are needed which should be at least stratified for folate levels and gender.

## Competing interest

The authors declare that they have no competing interests.

## Authors' contributions

HGW drafted the manuscript and performed data analysis. JLW edited the manuscript. HGW, JLW and LXZ participated in data collection. XCL and WJM conceived of the study and participated in designing the manuscript. All authors participated in revising the manuscript. All authors have read and approved the final manuscript.

## Pre-publication history

The pre-publication history for this paper can be accessed here:

http://www.biomedcentral.com/1471-2350/13/77/prepub

## Supplementary Material

Additional file 1 **Figure S1.Folate metabolism and the role of MTHFR. Modified from Wiemels et al. (5).** Click here for file

Additional file 2 **Table S1.General characteristics of studies included in the meta-analysis on* MTHFR *polymorphisms and ALL risk.** Click here for file

Additional file 3 **Table S2.*MTHFR*C677T genotype distribution and allele frequency in ALL cases and controls.** Click here for file

Additional file 4 **Table S3.*MTHFR*A1298C genotype distribution and allele frequency in ALL cases and controls.** Click here for file

Additional file 5 **Figure S2.Sensitive analysis to assess the influence of a single study in the meta-analysis on* MTHFR *C677T polymorphism and the risk of ALL.** Allele contrast was used in sensitive analysis. N, non-Caucasians, admixture of Amerindians, Europeans and Africans; W, mainly Brazilians of Caucasian descent. Click here for file

Additional file 6 **Figure S3.Sensitive analysis to assess the influence of a single study in the meta-analysis on* MTHFR *A1298C polymorphism and the risk of ALL.** Allele contrast was used in sensitive analysis. C, Chinese; M, Malays; N, non-Caucasians, admixture of Amerindians, Europeans and Africans; W, mainly Brazilians of Caucasian descent. Click here for file

Additional file 7 **Table S4.Results of the pooled OR and the corresponding 95% CIs for each combined genotype for* MTHFR *C677T and A1298C polymorphisms.** Click here for file

Additional file 8 **Table S5.Characteristics of meta-analyses regarding* MTHFR *polymorphisms and ALL risk.** (DOC 42 kb) Click here for file
