# Supplementary figures and images for: Methylenetetrahydrofolate Reductase Polymorphisms and Risk of Acute Lymphoblastic Leukemia-Evidence from an updated meta-analysis including 35 studies
Source: BMC Med Genet. 2012 Sep 4;13:77. doi: 10.1186/1471-2350-13-77 (PMC3459788; doi:10.1186/1471-2350-13-77)

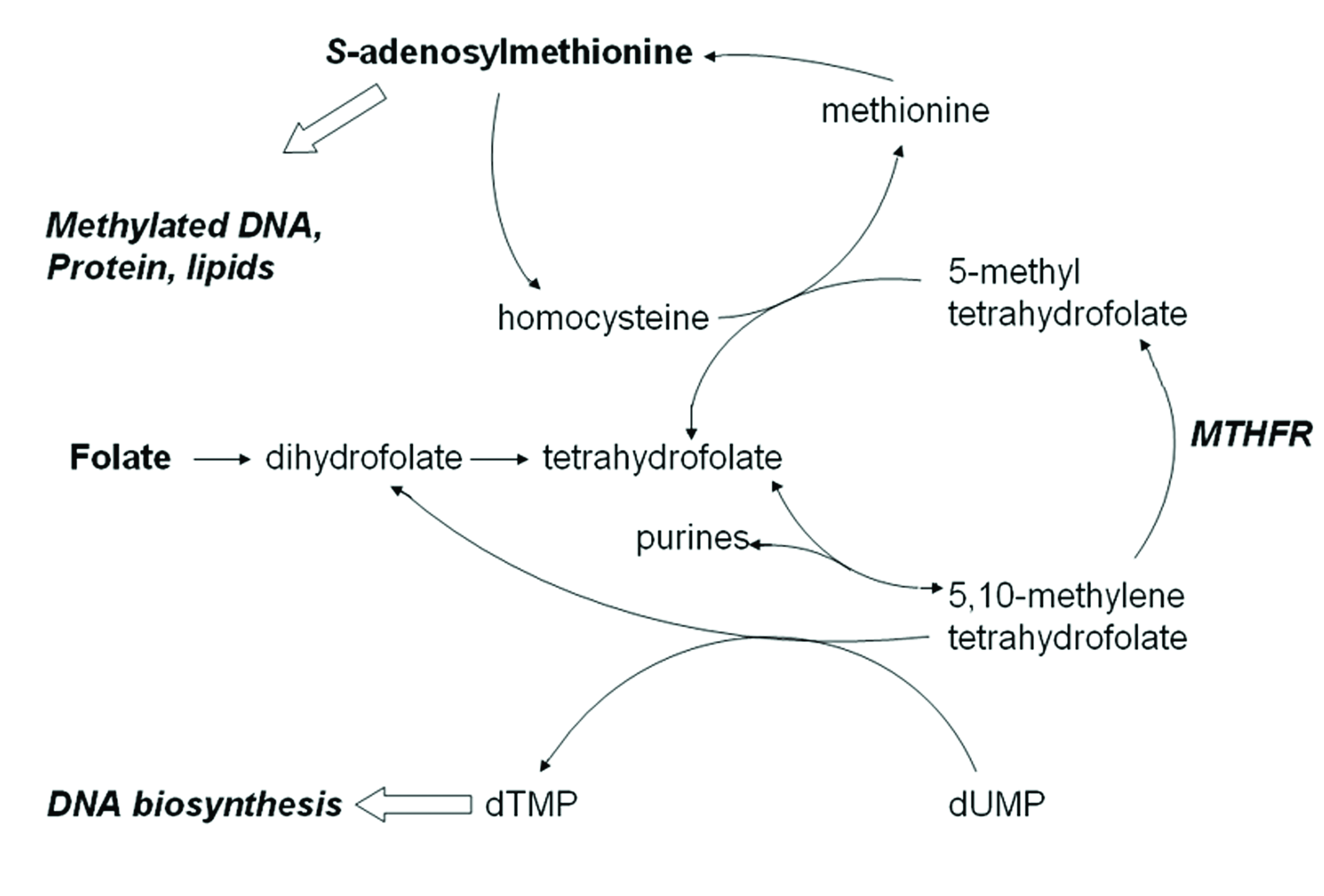

Supplement: Additional file 1 — Figure S1.Folate metabolism and the role of MTHFR. Modified from Wiemels et al. (5). [file 1471-2350-13-77-S1.tiff]

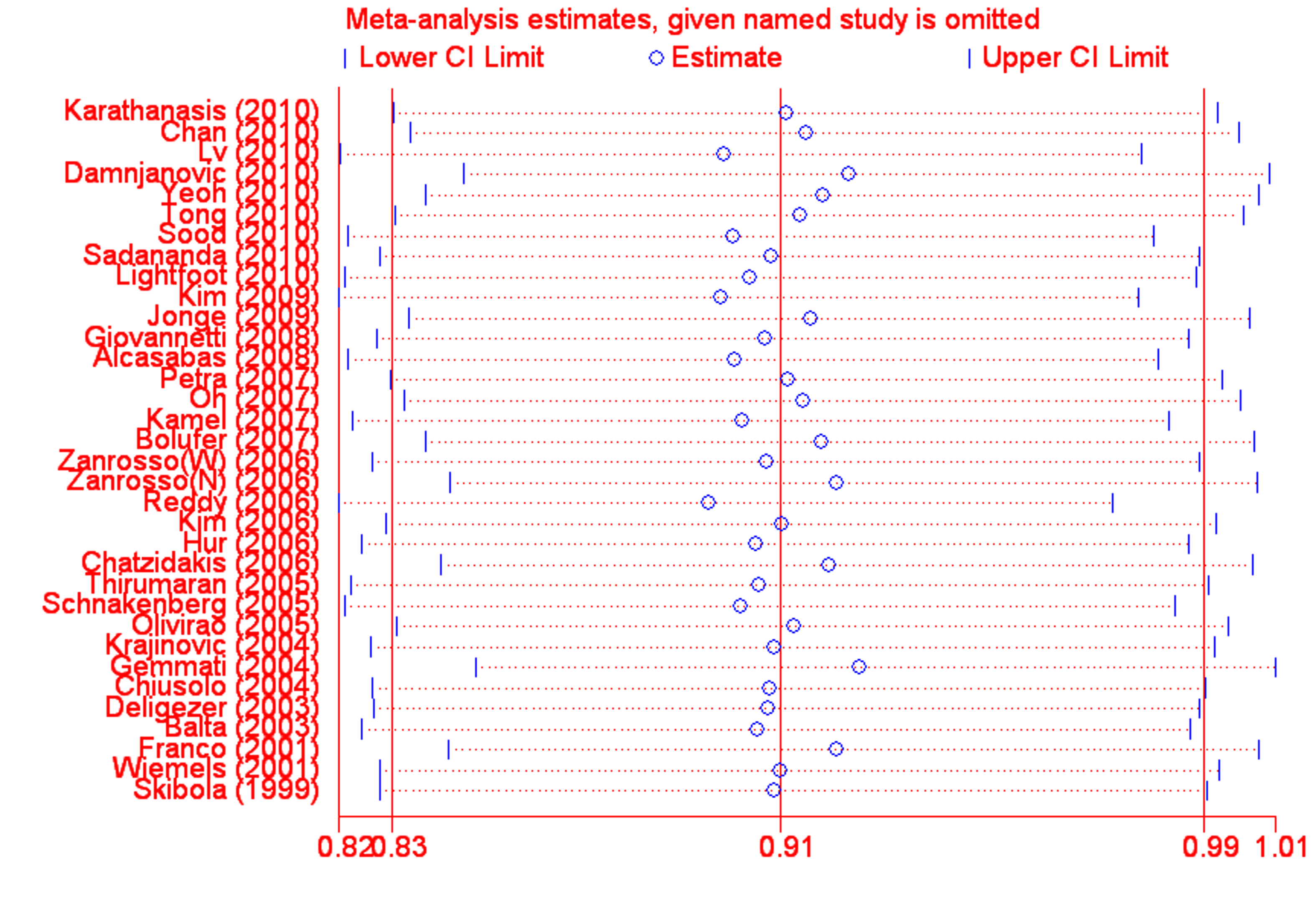

Supplement: Additional file 5 — Figure S2.Sensitive analysis to assess the influence of a single study in the meta-analysis on MTHFR C677T polymorphism and the risk of ALL. Allele contrast was used in sensitive analysis. N, non-Caucasians, admixture of Amerindians, Europeans and Africans; W, mainly Brazilians of Caucasian descent. [file 1471-2350-13-77-S5.tiff]

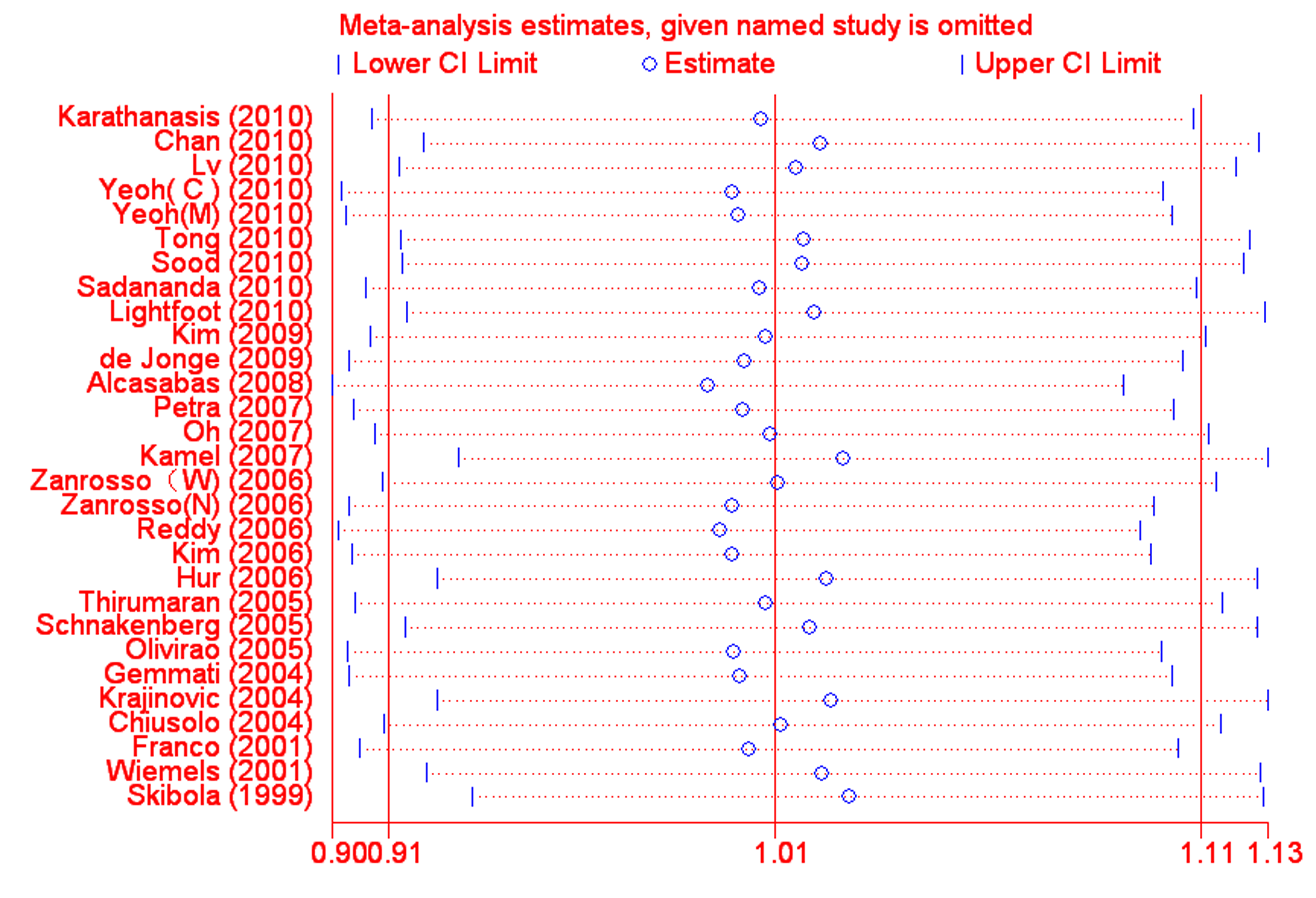

Supplement: Additional file 6 — Figure S3.Sensitive analysis to assess the influence of a single study in the meta-analysis on MTHFR A1298C polymorphism and the risk of ALL. Allele contrast was used in sensitive analysis. C, Chinese; M, Malays; N, non-Caucasians, admixture of Amerindians, Europeans and Africans; W, mainly Brazilians of Caucasian descent. [file 1471-2350-13-77-S6.tiff]
