# Supplementary material for: Methylenetetrahydrofolate Reductase Polymorphisms and Risk of Acute Lymphoblastic Leukemia-Evidence from an updated meta-analysis including 35 studies
Source: BMC Med Genet. 2012 Sep 4;13:77. doi: 10.1186/1471-2350-13-77 (PMC3459788; doi:10.1186/1471-2350-13-77)
Supplement: Additional file 2 — Table S1.General characteristics of studies included in the meta-analysis on MTHFR polymorphisms and ALL risk. [file 1471-2350-13-77-S2.doc]

**Supplement Table 1. General characteristics of studies included in the meta-analysis on *MTHFR* polymorphisms and ALL risk**

| **Source**  **(year)** | **Region/**  **country** | **No. of**  **cases (M/F)** | **No. of**  **controls (M/F)** | **Study population** | **Ethnicity** | **Source of controls** | **Genotyping**  **method** | **Subtypes of ALL^b^** | **Polymorphism(s) studied** |
| --- | --- | --- | --- | --- | --- | --- | --- | --- | --- |
| Skibola et al,^23^  (1999) | United Kingdom | 71(41/30) | 114(Nr) | Adults | Caucasians | Hospital | PCR-RFLP | 70.4% B-ALL, 15.5% T-ALL and 14.1% unspecified | C677T, A1298C |
| Wiemels  et al,^5^ (2001) | United Kingdom | 216(Nr) | 200(Nr) | Children | Mixed | Population | PCR-RFLP | Nr | C677T, A1298C |
| Franco et al,^24^  (2001) | Brazil | 71(20/51) | 71(20/51) | Children | Mixed | Nr | PCR-RFLP | 77.5% B-ALL, 22.5% T-ALL | C677T, A1298C |
| Deligezer  et al,^25^ (2003) | Turkey | 62(44/18) | 161(48/112) | Adults | Turkish | Nr | Real time PCR | Nr | C677T |
| Balta et al,^26^  (2003) | Turkey | 144(96/48) | 185(120/65) | Children | Turkish | Population | PCR-RFLP | 52.1% B-ALL, 28.5% T-ALL and 19.4% undetermined | C677T |
| Krajinovic  et al,^27^ (2004) | Canada | 270(157/113) | 300(Nr) | Children | Caucasians | Hospital | ASO | 84.4% B-ALL, 11.5% T-ALL and 4.1% undetermined | C677T, A1298C |
| Gemmati  et al,^28^ (2004) | Italy | 120(69/51) | 257(149/108) | Adults | Caucasians | Population | PCR-RFLP | 73.3% B-ALL, 16.6% T-ALL, and 10% undetermined | C677T, A1298C |
| Chiusolo  et al,^29^ (2004) | Italy | 174(96/78) | 110(75/35) | Children,  Adults | Caucasians | Population | PCR-RFLP | 90.2% B-ALL,  8.6% T-ALL, and 1.2% undetermined | C677T, A1298C |
| Schnakenberg  et al,^30^ (2005) | Germany, Austria and Switzerland | 433(268/175) | 379(Nr) | Children ^a^ | Caucasians | Population | Real time PCR | 77.7% B-ALL, 18.3% T-ALL, and 3.2% undetermined | C677T, A1298C |
| Oliveira  et al,^31^ (2005) | Portugal | 103(Nr) | 111(Nr) | Children ^a^ | Caucasians | Population | PCR-RFLP | Nr | C677T, A1298C |
| Thirumaran  et al,^32^ (2005) | Germany | 460(Nr) | 1472(Nr) | Children | Caucasians | Nr | Real time PCR | Nr | C677T, A1298C |
| Zanrosso  et al,^33^ (2006) | Brazil | 176(92/84) | 199(83/116) | Children | Caucasians,  and non- Caucasians | Population | PCR-RFLP | 94.9% B-ALL, 5.1% T-ALL | C677T, A1298C |
| Reddy et al,^34^  (2006) | India | 135(87/48) | 142(89/53) | Children | Indian | Hospital | PCR-RFLP | Nr | C677T, A1298C |
| Kim et al,^35^  (2006) | Korea | 66(39/27) | 100(Nr) | Children | Korean | Nr | ASO | Nr | C677T, A1298C |
| Hur et al,^36^  (2006) | Korea | 89(61/28) | 200(128/72) | Children,  Adults | Korean | Nr | PCR-RFLP | 88.8% B-ALL, 11.2% T-ALL | C677T, A1298C |
| Chatzidakis  et al,^37^ (2006) | Greece | 52(29/23) | 88(52/36) | Children ^a^ | Caucasians | Nr | PCR-RFLP | 86.5% B-ALL, 13.5% T-ALL | C677T |
| Petra et al,^38^  (2007) | Slovenia | 68(27/41) | 258(145/113) | Children ^a^ | Caucasians | Population | PCR-RFLP | 75.0% B-ALL, 11.8% T-ALL and 13.2% undetermined | C677T, A1298C |
| Oh et al,^39^  (2007) | Korea | 118(63/55) | 427(Nr) | Adults | Korean | Population | PCR-RFLP | Nr | C677T, A1298C |
| Kamel et al,^40^  (2007) | Egypt | 88(56/32) | 311(Nr) | Children ^a^ | Egyptian | Population | PCR-RFLP | 100% B-ALL | C677T, A1298C |
| Bolufer et al,^41^  (2007) | Spain | 141(87/54) | 454(223/231) | Children,  Adults | Caucasians | Population | Real-time PCR | 77.9% B-ALL, 16.3% T-ALL, and 5.6% Ph^+^-ALL | C677T |
| Giovannetti  et al,^42^ (2008) | Indonesia | 71(36/34) | 44(26/18) | Children | Indonesian | Hospital | Real time PCR | Nr | C677T |
| Alcasabas  et al,^43^ (2008) | Philippines | 191(115/76) | 394(209/185) | Children | Filipinos | Population | Real time PCR | Nr | C677T, A1298C |
| Kim et al,^44^  (2009) | Korea | 108(61/47) | 1700(821/879) | Adults | Korean | Population | PCR-RFLP, real-time PCR | Nr | C677T, A1298C |
| Jonge et al,^45^  (2009) | West Europe | 245(147/98) | 500 (Nr) | Children ^a^ | Caucasians | Population | PCR-RFLP | 75% B-ALL, 25% T-ALL | C677T, A1298C |
| Lv et al,^46^  (2010) | China | 127(59/68) | 182(85/97) | Adults | Chinese | Hospital | PCR-RFLP | 100% B-ALL | C677T, A1298C |
| Damnjanovic  et al,^47^ (2010) | Serbia | 78(Nr) | 412(Nr) | Children | Caucasians | Nr | PCR-RFLP | Nr | C677T |
| Yeoh et al,^48^  (2010) | Malaysia | 210(123/87) | 410(180/230) | Children | Malays | Nr | PCR-RFLP | 4.8% T-ALL, 90% B-ALL and 5.2% infant ALL | A1298C |
| Yeoh et al,^48^  (2010) | Singapore | 321(185/136) | 346(156/190) | Children | Chinese | Nr | PCR-RFLP | 7.8% T-ALL, 89.4% B-ALL and 2.8% infant ALL | C677T, A1298C |
| Tong et al,^49^  (2010) | China | 361(216/145) | 508(318/190) | Children | Chinese | Population | PCR-RFLP | 84.2 % B-ALL and 15.8% T-ALL | C677T, A1298C |
| Sood et al,^50^  (2010) | India | 95(70/25) | 255(Nr) | Children ^a^ | Indian | Population | PCR-RFLP | Nr | C677T, A1298C |
| Sadananda  et al,^51^ (2010) | India | 86(59/27) | 99(71/28) | Children | Indian | Population | PCR-RFLP | Nr | C677T, A1298C |
| Lightfoot  et al,^52^ (2010) | United Kingdom | 939(Nr) | 824(446/378) | Children | Caucasians | Population | Real time PCR | 81.5% B-ALL, 9.3% T-ALL and 9.3% undetermined | C677T, A1298C |
| Chan et al,^53^ (2010) | Indonesia | 185(107/78) | 177(104/73) | Children | Javanese | Population | PCR-RFLP | Nr | C677T, A1298C |
| Karathanasis  et al,^54^ (2011) | Greece | 35(21/14) | 48 | Children ^a^ | Caucasians | Population | PCR-RFLP | 88.6 % B-ALL and 11.4% T-ALL | C677T, A1298C |

ASO= allele-specific oligonucleotide hybridization; F= females; M= males; Nr= not report.

^a^ Control group composed of adults.

^b^ The subtypes of ALL were determined based on immunophenotypic studies.
