# Supplementary material for: Methylenetetrahydrofolate Reductase Polymorphisms and Risk of Acute Lymphoblastic Leukemia-Evidence from an updated meta-analysis including 35 studies
Source: BMC Med Genet. 2012 Sep 4;13:77. doi: 10.1186/1471-2350-13-77 (PMC3459788; doi:10.1186/1471-2350-13-77)
Supplement: Additional file 3 — Table S2.MTHFRC677T genotype distribution and allele frequency in ALL cases and controls. [file 1471-2350-13-77-S3.doc]

**Supplement Table 2.** ***MTHFR* C677T genotype distribution and allele frequency in ALL cases and controls**

| **Source (year)** | **Cases** | | | |  | **Controls** | | | |  | **HWE** |
| --- | --- | --- | --- | --- | --- | --- | --- | --- | --- | --- | --- |
|  | **CC** | **CT** | **TT** | **T** |  | **CC** | **CT** | **TT** | **T** |  |  |
| Skibola et al,^23^ (1999) | 35(51) | 29(42) | 5(7) | 39(28) |  | 61(54) | 39(34) | 14(12) | 67(29) |  | 0.06 |
| Wiemels et al,^5^ (2001) | 98(45) | 91(42) | 27(13) | 145(34) |  | 88(44) | 79(40) | 32(16) | 143(36) |  | 0.05 |
| Franco et al,^24^ (2001) | 36(51) | 28(40) | 6(9) | 40(29) |  | 22(31) | 36(51) | 13(18) | 62(44) |  | 0.25 |
| Deligezer et al,^25^ (2003) | 27(44) | 31(50) | 4(6) | 39(31) |  | 74(46) | 73(45) | 14(9) | 101(31) |  | 0.50 |
| Balta et al,^26^ (2003) | 71(50) | 60(42) | 11(8) | 82(29) |  | 90(49) | 87(47) | 8(4) | 103(28) |  | 0.02 |
| Krajinovic et al,^27^ (2004) | 112(41) | 127(47) | 31(11) | 189(35) |  | 126(42) | 128(43) | 46(15) | 220(37) |  | 0.16 |
| Gemmati et al,^28^ (2004) | 52(46) | 53(46) | 9(8) | 71(31) |  | 78(30) | 128(50) | 51(20) | 230(45) |  | 0.91 |
| Chiusolo et al,^29^ (2004) | 65(37) | 71(41) | 38(22) | 147(42) |  | 35(32) | 55(50) | 20(18) | 95(43) |  | 0.84 |
| Schnakenberg et al,^30^ (2005) | 195(44) | 201(45) | 47(11) | 295(33) |  | 184(49) | 152(40) | 43(11) | 238(31) |  | 0.18 |
| Oliveira et al,^31^ (2005) | 48(47) | 50(49) | 5(5) | 60(29) |  | 45(41) | 57(51) | 9(8) | 75(34) |  | 0.12 |
| Thirumaran et al,^32^ (2005) | 199(44) | 195(43) | 59(13) | 313(35) |  | 600(41) | 681(47) | 167(12) | 1015(35) |  | 0.21 |
| Zanrosso et al (W),^33^ (2006) | 43(50) | 35(41) | 8(9) | 51(30) |  | 59(50) | 50(42) | 10(8) | 70(29) |  | 0.90 |
| Zanrosso et al (N),^33^ (2006) | 53(67) | 21(27) | 5(6) | 31(20) |  | 37(47) | 32(41) | 10(13) | 52(33) |  | 0.46 |
| Reddy et al,^34^ (2006) | 51(38) | 77(57) | 7(5) | 91(34) |  | 79(56) | 58(41) | 5(4) | 68(24) |  | 0.15 |
| Kim et al,^35^ (2006) | 17(26) | 38(58) | 11(17) | 60(45) |  | 24(24) | 55(55) | 21(21) | 97(49) |  | 0.31 |
| Hur et al,^36^ (2006) | 30(34) | 44(59) | 15(17) | 74(42) |  | 80(40) | 80(40) | 40(20) | 160(40) |  | 0.02 |
| Chatzidakis et al,^37^ (2006) | 31(60) | 18(35) | 3(6) | 24(23) |  | 32(36) | 47(53) | 9(10) | 65(37) |  | 0.17 |
| Petra et al,^38^ (2007) | 30(44) | 33(49) | 5(7) | 43(32) |  | 112(43) | 110(43) | 36(14) | 182(35) |  | 0.29 |
| Oh et al,^39^ (2007) | 49(42) | 55(47) | 14(12) | 83(35) |  | 138(32) | 229(54) | 60(14) | 349(41) |  | 0.02 |
| Kamel et al,^40^ (2007) | 39(44) | 42(48) | 7(8) | 56(32) |  | 156(50) | 135(43) | 20(6) | 175(28) |  | 0.92 |
| Bolufer et al,^41^ (2007) | 49(42) | 53(45) | 15(13) | 83(35) |  | 106(32) | 160(48) | 65(20) | 290(44) |  | 0.74 |
| Giovannetti et al,^42^ (2008) | 51(78) | 11(17) | 3(5) | 17(13) |  | 26(81) | 6(19) | 0(0) | 6(9) |  | 0.56 |
| Alcasabas et al,^43^ (2008) | 145(77) | 41(22) | 3(2) | 47(12) |  | 322(82) | 66(17) | 6(2) | 78(10) |  | 0.23 |
| Kim et al,^44^ (2009) | 29(27) | 51(48) | 27(25) | 105(49) |  | 540(32) | 863(51) | 297(17) | 1457(17) |  | 0.13 |
| Jonge et al,^45^ (2009) | 130(53) | 93(38) | 22(9) | 137(28) |  | 219(44) | 223(45) | 54(11) | 331(33) |  | 0.81 |
| Lv et al,^46^ (2010) | 38(30) | 65(51) | 24(19) | 113(44) |  | 72(40) | 83(46) | 27(15) | 137(38) |  | 0.70 |
| Damnjanovic et al,^47^ (2010) | 45(58) | 28(36) | 5(6) | 38(24) |  | 163(40) | 190(46) | 59(14) | 308(37) |  | 0.76 |
| Yeoh et al,^48^ (2010) | 184(58) | 111(35) | 23(7) | 157(25) |  | 163(47) | 150(43) | 32(9) | 214(31) |  | 0.77 |
| Tong et al,^49^ (2010) | 135(37) | 192(53) | 34(9) | 260(36) |  | 173(34) | 257(51) | 78(15) | 413(41) |  | 0.27 |
| Sood et al,^50^ (2010) | 54(57) | 38(40) | 3(3) | 44(23) |  | 173(68) | 71(28) | 11(4) | 93(18) |  | 0.29 |
| Sadananda et al,^51^ (2010) | 73(85) | 13(15) | 0(0) | 13(8) |  | 85(86) | 14(14) | 0(0) | 14(7) |  | 0.45 |
| Lightfoot et al,^52^ (2010) | 374(46) | 341(42) | 90(11) | 521(32) |  | 359(47) | 317(42) | 84(11) | 485(32) |  | 0.27 |
| Chan et al,^53^ (2010) | 140(76) | 43(23) | 2(1) | 47(13) |  | 122(69) | 51(29) | 4(2) | 59(33) |  | 0.62 |
| Karathanasis et al,^54^ (2011) | 17(49) | 13(37) | 5(14) | 23(33) |  | 18(38) | 24(50) | 6(13) | 36(38) |  | 0.64 |

HWE=Hardy–Weinberg equilibrium; N=non-Caucasians, admixture of Amerindians, Europeans and Africans; W=mainly Brazilians of Caucasian descent.
