# Supplementary material for: Methylenetetrahydrofolate Reductase Polymorphisms and Risk of Acute Lymphoblastic Leukemia-Evidence from an updated meta-analysis including 35 studies
Source: BMC Med Genet. 2012 Sep 4;13:77. doi: 10.1186/1471-2350-13-77 (PMC3459788; doi:10.1186/1471-2350-13-77)
Supplement: Additional file 4 — Table S3.MTHFRA1298C genotype distribution and allele frequency in ALL cases and controls. [file 1471-2350-13-77-S4.doc]

**Supplement Table 3. *MTHFR* A1298C genotype distribution and allele frequency in ALL** cases and controls

| **Source (year)** | **Cases** | | | |  | **Controls** | | | |  | **HWE** |
| --- | --- | --- | --- | --- | --- | --- | --- | --- | --- | --- | --- |
|  | **AA** | **AC** | **CC** | **C** |  | **AA** | **AC** | **CC** | **C** |  |  |
| Skibola et al,^23^ (1999) | 45(65) | 23(33) | 1(1) | 25(18) |  | 49(43) | 54(47) | 11(10) | 76(33) |  | 0.48 |
| Wiemels et al,^5^ (2001) | 109(52) | 90(43) | 11(5) | 112(27) |  | 93(47) | 83(42) | 23(12) | 129(32) |  | 0.50 |
| Franco et al,^24^ (2001) | 36(51) | 30(42) | 5(7) | 40(28) |  | 41(58) | 28(39) | 2(3) | 32(23) |  | 0.28 |
| Krajinovic et al,^27^ (2004) | 151(56) | 107(40) | 12(4) | 131(24) |  | 150(50) | 119(40) | 31(10) | 181(30) |  | 0.31 |
| Gemmati et al,^28^ (2004) | 47(41) | 55(48) | 12(11) | 79(35) |  | 126(49) | 110(43) | 21(8) | 152(30) |  | 0.66 |
| Chiusolo et al,^29^ (2004) | 92(53) | 73(48) | 9(5) | 91(26) |  | 56(51) | 49(45) | 5(5) | 59((27) |  | 0.16 |
| Schnakenberg et al,^30^ (2005) | 194(44) | 204(46) | 45(10) | 294(33) |  | 153(40) | 174(46) | 52(14) | 278(37) |  | 0.82 |
| Oliveira et al,^31^ (2005) | 36(35) | 58(56) | 9(9) | 76(37) |  | 54(49) | 48(43) | 9(8) | 66(30) |  | 0.71 |
| Thirumaran et al,^32^ (2005) | 198(44) | 195(44) | 52(12) | 299(34) |  | 660(45) | 644(44) | 149(10) | 942(32) |  | 0.66 |
| Zanrosso et al (W),^33^ (2006) | 48(53) | 35(39) | 7(8) | 49(27) |  | 62(52) | 50(42) | 8(7) | 66(28) |  | 0.62 |
| Zanrosso et al (N),^33^ (2006) | 35(45) | 39(50) | 4(5) | 47(30) |  | 49(62) | 26(33) | 4(5) | 34(22) |  | 0.82 |
| Reddy et al,^34^ (2006) | 41(30) | 83(61) | 11(8) | 105(39) |  | 65(46) | 68(48) | 9(6) | 86(30) |  | 0.11 |
| Kim et al,^35^ (2006) | 38(61) | 23(37) | 1(2) | 25(20) |  | 77(77) | 21(21) | 2(2) | 25(13) |  | 0.69 |
| Hur et al,^36^ (2006) | 64(72) | 23(26) | 2(2) | 27(15) |  | 116(58) | 78(39) | 6(3) | 90(23) |  | 0.09 |
| Petra et al,^38^ (2007) | 27(40) | 32(47) | 9(13) | 50(37) |  | 129(50) | 97(38) | 32(12) | 161(31) |  | 0.05 |
| Oh et al,^39^ (2007) | 72(67) | 33(31) | 2(2) | 37(17) |  | 293(69) | 126(30) | 8(2) | 142(17) |  | 0.18 |
| Kamel et al,^40^ (2007) | 58(66) | 22(25) | 8(9) | 38(22) |  | 141(45) | 140(45) | 29(9) | 198(32) |  | 0.49 |
| Alcasabas et al,^43^ (2008) | 56(30) | 94(50) | 38(20) | 170(45) |  | 160(41) | 178(45) | 56(14) | 290(37) |  | 0.57 |
| Kim et al,^44^ (2009) | 67(63) | 38(36) | 1(1) | 40(19) |  | 1147(67) | 500(29) | 53(3) | 606(18) |  | 0.87 |
| Jonge et al,^45^ (2009) | 110(45) | 100(41) | 35(14) | 170(35) |  | 229((47) | 213(44) | 45(9) | 303(31) |  | 0.65 |
| Lv et al,^46^ (2010) | 86(34) | 36(28) | 5(4) | 46(18) |  | 111(61) | 67(37) | 4(2) | 75(21) |  | 0.09 |
| Yeoh et al(C),^48^ (2010) | 174(55) | 121(38) | 23(7) | 167(26) |  | 211(61) | 115(33) | 18(5) | 151(22) |  | 0.65 |
| Yeoh et al(M),^48^ (2010) | 85(41) | 97(47) | 26(13) | 149(36) |  | 157(49) | 130(40) | 36(11) | 202(31) |  | 0.25 |
| Tong et al,^49^ (2010) | 256(71) | 90(25) | 15(4) | 120(17) |  | 342(67) | 142(28) | 24(5) | 190(19) |  | 0.07 |
| Sood et al,^50^ (2010) | 30(32) | 46(48) | 19(20) | 84(44) |  | 59(23) | 146(57) | 50(20) | 246(48) |  | 0.02 |
| Sadananda et al,^51^ (2010) | 20(23) | 51(59) | 15(17) | 81(47) |  | 28(28) | 55(56) | 16(16) | 87(44) |  | 0.20 |
| Lightfoot et al,^52^ (2010) | 408(52) | 305(39) | 73(9) | 451(29) |  | 350(46) | 332(44) | 77(10) | 486(32) |  | 0.89 |
| Chan et al,^53^ (2010) | 99(54) | 73(40) | 13(7) | 99(27) |  | 82(46) | 75(42) | 20(11) | 115(32) |  | 0.65 |
| Karathanasis et al,^54^ (2011) | 14(40) | 17(49) | 4(11) | 25(36) |  | 22(46) | 22(46) | 4(8) | 30(31) |  | 0.64 |

C=Chinese; HWE=Hardy–Weinberg equilibrium; M=Malays; N=non-Caucasians, admixture of Amerindians, Europeans and Africans; W=mainly Brazilians of Caucasian descent.
