# Supplementary material for: Methylenetetrahydrofolate Reductase Polymorphisms and Risk of Acute Lymphoblastic Leukemia-Evidence from an updated meta-analysis including 35 studies
Source: BMC Med Genet. 2012 Sep 4;13:77. doi: 10.1186/1471-2350-13-77 (PMC3459788; doi:10.1186/1471-2350-13-77)
Supplement: Additional file 7 — Table S4.Results of the pooled OR and the corresponding 95% CIs for each combined genotype for MTHFR C677T and A1298C polymorphisms. [file 1471-2350-13-77-S7.doc]

**Supplement Table 4. Results of the pooled OR and the corresponding 95% CIs for each combined genotype for *MTHFR* C677T and A1298C polymorphisms**

|  | OR | 95%CIs |
| --- | --- | --- |
| CC/AA | Baseline | Baseline |
| CC/AC | 0.88 | 0.66-1.17 |
| CC/CC | 0.97 | 0.62-1.54 |
| CT/AA | 1.06 | 0.87-1.29 |
| CT/AC | 1.14 | 0.78-1.68 |
| CT/CC | 1.14 | 0.48-2.72 |
| TT/AA | 0.90 | 0.68-1.18 |
| TT/AC | 1.36 | 0.35-5.30 |
