# Supplementary material for: Methylenetetrahydrofolate Reductase Polymorphisms and Risk of Acute Lymphoblastic Leukemia-Evidence from an updated meta-analysis including 35 studies
Source: BMC Med Genet. 2012 Sep 4;13:77. doi: 10.1186/1471-2350-13-77 (PMC3459788; doi:10.1186/1471-2350-13-77)
Supplement: Additional file 8 — Table S5.Characteristics of meta-analyses regarding MTHFR polymorphisms and ALL risk. (DOC 42 kb) [file 1471-2350-13-77-S8.doc]

**Supplement Table 5. Characteristics of meta-analyses regarding *MTHFR* polymorphisms and ALL risk**

| **Author**  **(year)** | **Polymorphisms studied** | **Study population** | **Included studies** | **Subgroup**  **analysis** | **Meta-regression**  **analysis** | **Cumulative and recursive cumulative meta-analyses** | **Pooled OR** |
| --- | --- | --- | --- | --- | --- | --- | --- |
| Pereira  et al,^12^ (2006) | C677T,  A1298C | Adults  Children | 13 (C677T)  11 (A1298C) | Children | For A1298C polymorphism, no study-level covariates were found | Not done. | C677T:  0.83(0.70-0.99)^b^  A1298C:  0.83(0.60-1.15)^b^ |
| Zintzaras et al,^13^  (2006) | C677T,  A1298C | Adults  Children | 9 (C677T)  7 (A1298C) | Caucasians  Children | Not done. | The result showed the OR was not stable. | C677T: 0.88(0.76-1.02)^a^  A1298C:  0.67(0.46-0.99)^b^ |
| Koppen et al,^14^  (2009) | C677T,  A1298C | Children | 7 (C677T)  6 (A1298C) | Not done | Not done | Not done | C677T:  0.76(0.63-0.91)^c^  A1298C:  0.84(0.59-1.21)^c^ |
| Wang et al,^15^ (2010) | C677T | Children | 18 | Whites  Europeans  Asians  East Asians | Not done | Not done | C677T:  0.93(0.82-1.07)^a^ |
| Zacho et al,^16^ (2011) | C677T | Adults  Children | 37^d^ | Not done | Not done | Not done | C677T:  0.84(0.74-0.96)^c^ |
| Yan et al,^17^  (2011) | C677T,  A1298C | Children | 21 (C677T)  17 (A1298C) | Caucasians  Asians | Not done | Not done | C677T:  0.83(0.72-0.95)^b^  A1298C:  1.02(0.89-1.17)^b^ |
| Current study | C677T,  A1298C | Adults  Children | 34 (C677T)  29 (A1298C) | Caucasians  East Asians  Adults  Children | M/F in cases could modify the C677T variant effects in relation to ALL | The result showed that ORs for the C677T stabilized as evidence accumulated. | C677T:  0.91(0.83-0.99)^a^  A1298C:  1.01(0.91-1.11)^a^ |

^a^ Comparison by the allele contrast.

^b^ Comparison by recessive genetic model.

^c^ Comparison model was not reported.

^d^ acute lymphatic leukemia, acute myeloid leukemia, chronic lymphatic leukemia and chronic myeloid leukemia were included.
